# Supplementary material for: Content-rich biological network constructed by mining PubMed abstracts
Source: BMC Bioinformatics. 2004 Oct 8;5:147. doi: 10.1186/1471-2105-5-147 (PMC528731; doi:10.1186/1471-2105-5-147)
Supplement: Additional File 2 — The original results of the above study (non-essential files are deleted to keep the file size under the limit set by BMC bioinformatics). [file 1471-2105-5-147-S2.bz2 › chilibotAdditionalFile2/dip10/687ID11983079E18006/html/ISCS_NIFU.html]

 


 **ISCS** and **NIFU** 
  
Found 7 abstracts in PubMed, retrieved 7.  
 

 What does Google say? 
 PDF only 
| .edu only 

---

**Interactive relationship** (e.g. stimulation, inhibition, etc)

**Non-interactive relationship** (e.g. studied together, co-existance, homology, etc.)

- Because homologs to both  **NifU**  and NifS, respectively designated IscU and  **IscS** , are found in non nitrogen fixing organisms, it is possible that the function of  **NifU**  proposed here could represent a general mechanism for the maturation of Fe S cluster containing proteins.  Ref: 10639125 Proc Natl Acad Sci U S A, 2000
- **NifU**  and IscU have been proposed to serve as template or scaffold proteins in the initial Fe S cluster assembly events, but the mechanism of sulfur transfer from NifS or  **IscS**  to  **NifU**  or IscU has not been elucidated.  Ref: 11577100 J Biol Chem, 2001
- The  **iscS**  gene is contained within a gene cluster that includes homologs to  **nifU**  and another gene contained within the major nif cluster of A. vinelandii previously designated orf6.  Ref: 9582371 J Biol Chem, 1998
